# Direct and indirect cardiovascular and cardiometabolic sequelae of the combined anti-retroviral therapy on people living with HIV

**DOI:** 10.3389/fphys.2023.1118653

**Published:** 2023-03-27

**Authors:** Yashvardhan Batta, Cody King, Farion Cooper, John Johnson, Natasha Haddad, Myriam G. Boueri, Ella DeBerry, Georges E. Haddad

**Affiliations:** ^1^ Department of Physiology and Biophysics, College of Medicine, Howard University, Washington, DC, United States; ^2^ Delaware Psychiatric Center, New Castle, DE, United States; ^3^ Medical School, Lebanese American University, Beirut, Lebanon

**Keywords:** combined anti-retroviral treatment (cART), HIV, highly active anti-retroviral therapy (HAART), cardiovascular disease, cardiometabolic dysfunction, Neurology

## Abstract

With reports of its emergence as far back as the early 1900s, human immunodeficiency virus (HIV) has become one of the deadliest and most difficult viruses to treat in the era of modern medicine. Although not always effective, HIV treatment has evolved and improved substantially over the past few decades. Despite the major advancements in the efficacy of HIV therapy, there are mounting concerns about the physiological, cardiovascular, and neurological sequelae of current treatments. The objective of this review is to (Blattner et al., Cancer Res., 1985, 45(9 Suppl), 4598s–601s) highlight the different forms of antiretroviral therapy, how they work, and any effects that they may have on the cardiovascular health of patients living with HIV, and to (Mann et al., J Infect Dis, 1992, 165(2), 245–50) explore the new, more common therapeutic combinations currently available and their effects on cardiovascular and neurological health. We executed a computer-based literature search using databases such as PubMed to look for relevant, original articles that were published after 1998 to current year. Articles that had relevance, in any capacity, to the field of HIV therapy and its intersection with cardiovascular and neurological health were included. Amongst currently used classes of HIV therapies, protease inhibitors (PIs) and combined anti-retroviral therapy (cART) were found to have an overall negative effect on the cardiovascular system related to increased cardiac apoptosis, reduced repair mechanisms, block hyperplasia/hypertrophy, decreased ATP production in the heart tissue, increased total cholesterol, low-density lipoproteins, triglycerides, and gross endothelial dysfunction. The review of Integrase Strand Transfer Inhibitors (INSTI), Nucleoside Reverse Transcriptase Inhibitors (NRTI), and Non-Nucleoside Reverse Transcriptase Inhibitors (NNRTI) revealed mixed results, in which both positive and negative effects on cardiovascular health were observed. In parallel, studies suggest that autonomic dysfunction caused by these drugs is a frequent and significant occurrence that needs to be closely monitored in all HIV + patients. While still a relatively nascent field, more research on the cardiovascular and neurological implications of HIV therapy is crucial to accurately evaluate patient risk.

## Introduction

Among the plethora of viral diseases, HIV (human immunodeficiency virus) infection has always been one of the most widespread. In 1985, it was referred to as a pandemic for the first time (Blattner et al., 1985) which was shortly later supported by many other scientists (Richards et al., 1985; Mann, 1987; Li et al., 1988; Mann and Kay, 1991; Mann, 1992; De Cock et al., 2011; Tran et al., 2019). The emergence of this pandemic illness dates to the early 1900s as a zoonotic infection from chimpanzees of Central and West Africa. Although the virus is most often contracted from unprotected vaginal or anal sex, transmission may also occur from sharing needles or open cut exposure to HIV-infected bodily fluids. The virus targets and depletes CD4^+^ T lymphocytes, thereby compromising immune function. Untreated, infection with this retrovirus may eventually progress to a later third stage of illness, commonly referred to as AIDS (acquired immunodeficiency syndrome). With over 1.1 million people in the US currently living with a diagnosis of HIV, nearly 38,000 more get diagnosed every year (Scaccia, 2020). From the initial treatments in the 1980s, triple therapy cocktails in the 1990s, to the antiretroviral therapies given today, treatment for HIV/AIDS has favorably evolved and improved outcomes. In 2004, AIDS-related deaths peaked at nearly 2 million in 2004 and have now fallen to 680,000 in 2020. In 1996, the life expectancy of a 20-year-old person/people living with HIV (PLHIV) was 39 years. In 2011, that life expectancy rose to 70 years (Scaccia, 2020).

Evidently, such statistics point to a bettered long-term outlook of people living with HIV. Over the past couple of decades, HIV has transitioned from being viewed as an acute and fatal illness to a chronic disease of immunodeficiency. Its chronic management and treatment suppress the viral load to approximately 50 copies/mL, thereby reducing progression to AIDS. Not only does this prevent disease progression, but it also reduces chances of transmission from person-to-person. The problem remains to provide global coverage of these newly developed therapies.

**FIGURE 1:**
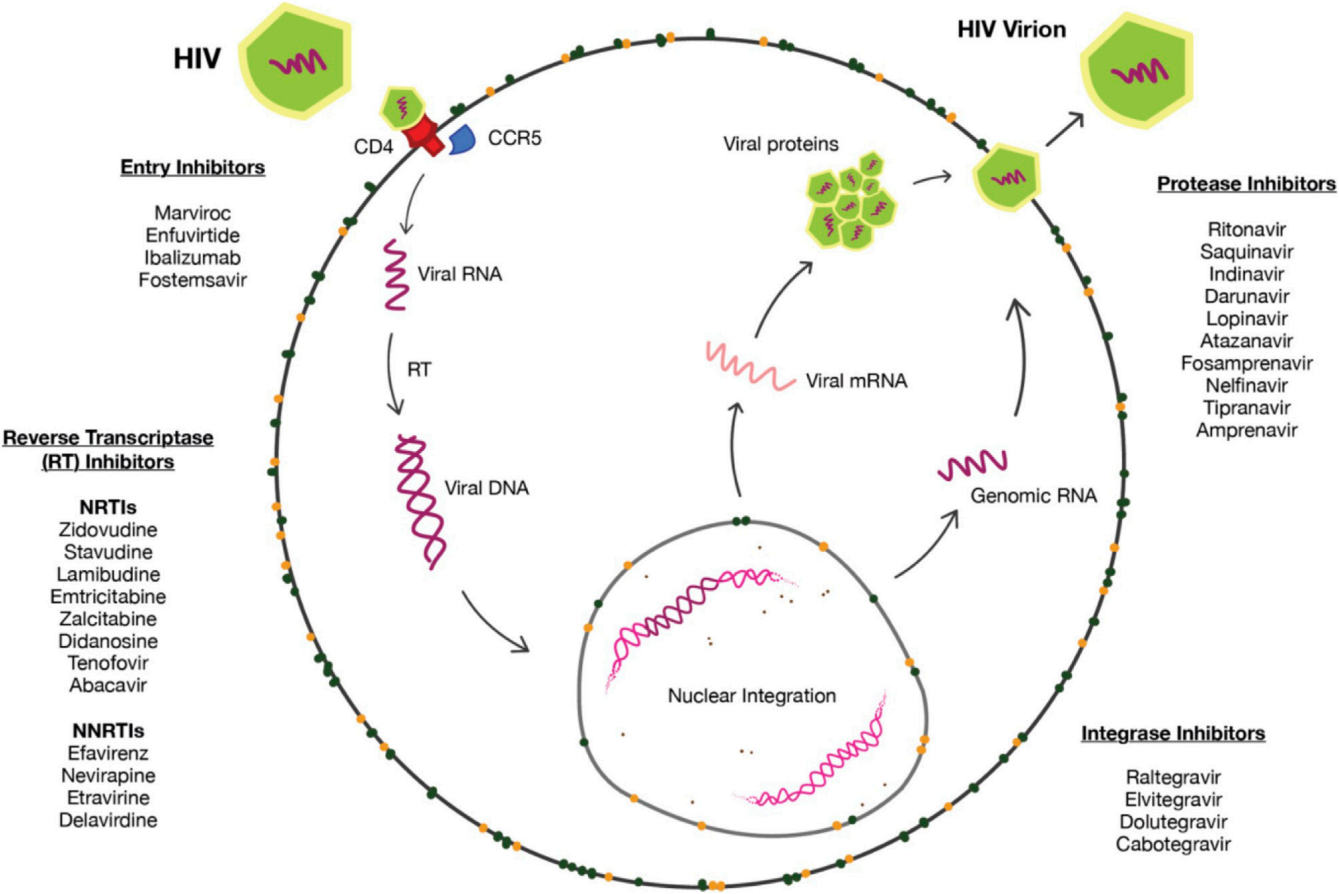
HIV pharmacological targets.

Decades after the initial reports, the global spread of HIV has turned this epidemic, as so often referred, into a modern pandemic with a disproportionate skew in impoverished communities (Staupe-Delgado and Rubin, 2022) In 2021, 38.4 million people were living with HIV worldwide. Another 1.5 million became newly infected while 650000 passed due to AIDS-related disease. A majority (74.7%) of those with HIV worldwide had access to antiretroviral therapy (FACT Sheet, 2022). These global HIV/AIDS statistics indicate that although advancements have been made, a greater effort is still needed to tackle health disparities and ensure widespread access to these medications. As the medical and global health communities aim to minimize these treatment gaps, further research into various treatment regimes has allowed a plethora of options in treating and managing the chronicity of HIV/AIDS. Several drug classes have been developed with unique mechanisms of action. From protease inhibitors, integrase strand transfer inhibitors, nucleoside reverse transcriptase inhibitors, to non-nucleoside reverse transcriptase inhibitors, each works to deplete the body of viral load (Figure 1). As will be extensively discussed in this review, their respective modes of action can be overlapped and merged to optimize treatment in the form of combination anti-retroviral therapy (cART).

This review aims to discuss scientific literature from 1988 to current years while highlighting the different classes of medications in HIV treatment. The authors hope to highlight the different forms of cART and discuss the mechanisms by which they exert their effects. Although PLHIV live longer and relatively healthier life as compared to their counterparts from the 20th century, it begs the question of its age-related cardiovascular impact along with its effects on the autonomic nervous system. This analysis will be done with an emphasis on the cardiovascular and some neurological implications of this chronic disease and treatment. Research in the field of HIV treatment is ever-growing. Although cART can reduce viral loads in patients, newly engineered CRISP/Cas-9 therapy uses gene editing technology to target the HIV-1 genome, cellular factors, and provirus directly. Off-target effects remain an important limitation. Still, application of this new modality holds for strong potential (Xiao et al., 2019). Further studies also highlight the role of hematopoietic stem cell transplantation (HSCT) for the treatment and potential cure of HIV infection. This 9-year study follows a 53-year-old PLHIV treated with HSCT for acute myeloid leukemia and indicated no viral rebound or HIV-1 antigen persistence (Jensen et al., 2023).

While cART has played a pivotal role in HIV management, evidence shows that it may negatively impact cardiovascular health (Table 1). The associated decline in cardiovascular health is so pervasive that patients receiving abacavir (a nucleoside reverse transcriptase inhibitor) were found to be at twice the risk of developing coronary vascular disease, as compared to the untreated group (Antony et al., 2020). Additionally, Obel et al. (2010) (Obel et al., 2010) suggested that patients actively receiving cART have an increased risk of ischemic heart disease when compared with the general population. According to Grinspoon et al. (2008) (Grinspoon et al., 2008), cART may also impact embryonic development of the cardiovascular system. *In utero* exposure is linked with a reduction in left ventricle mass and a decrease in septal wall thickness in the first year after birth. The incidence of coronary heart disease rose noticeably with exposure to cART, especially those containing protease inhibitors compared to those that contained non-nucleoside reverse transcriptase inhibitors (relative risk of 1.16 vs 1.05 respectively). Dorjee et al. (2021) (Dorjee et al., 2021) further found that tenofovir-emtricitabine-raltegravir is associated with an increased risk of acute myocardial infarction. However, this association was heavily questioned as these PLHIV were found to have an adverse cardiovascular disease risk prior to receiving cART.

**TABLE 1:** Individual Drug Classes and Cardiovascular Health Risk.

Drug	Cardiovascular Effects	References
*Protease Inhibitors*	• Increased cholesterol and triglyceride levels • Increased risk of atherosclerosis Increased risk myocardial infarction	Fontas et al., 2020 Zhou et al., 2005 Seminari et al., 2002 Mercie et al., 2002 DAD Study Group, 2007
*Integrase Inhibitors*	• Lower risk of cardiovascular disease and major cardiac events	O’Halloran et al., 2020 Fichtenbaum et al., 2010 Antony et al., 2020
*Nucleoside Reverse Transcriptase Inhibitors*	• Elevated lipid levels, cholesterol, and triglycerides • Increased risk of cardiovascular disease • Increase risk myocardial infarction	Antony et al., 2020 Islam et al., 2012 DAD Study Group et al., 2008 Lang et al., 2010 Obel et al., 2010 Ewayo et al., 2019 Durand et al., 2011 Worm et al., 2010 Bavinger et al., 2013
*Non-Nucleoside Reverse Transcriptase Inhibitors*	• Improved lipid and glucose profiles • No significant association with cardiovascular risk • Increased endothelial permeability • Increased risk myocardial infarction	Maggi et al., 2011 Fisac et al. (2003) DAD Study Group et al., 2007 Worm et al., 2010 Silverberg et al., 2014 Islam et al., 2012 Eyawo et al., 2012 Lang et al., 2010 Rosenblatt et al., 2016 Durand et al., 2011

Green = Beneficial Effects, Red = Harmful Effects, Gray = No Significant change/Neutral.

**FIGURE 2:**
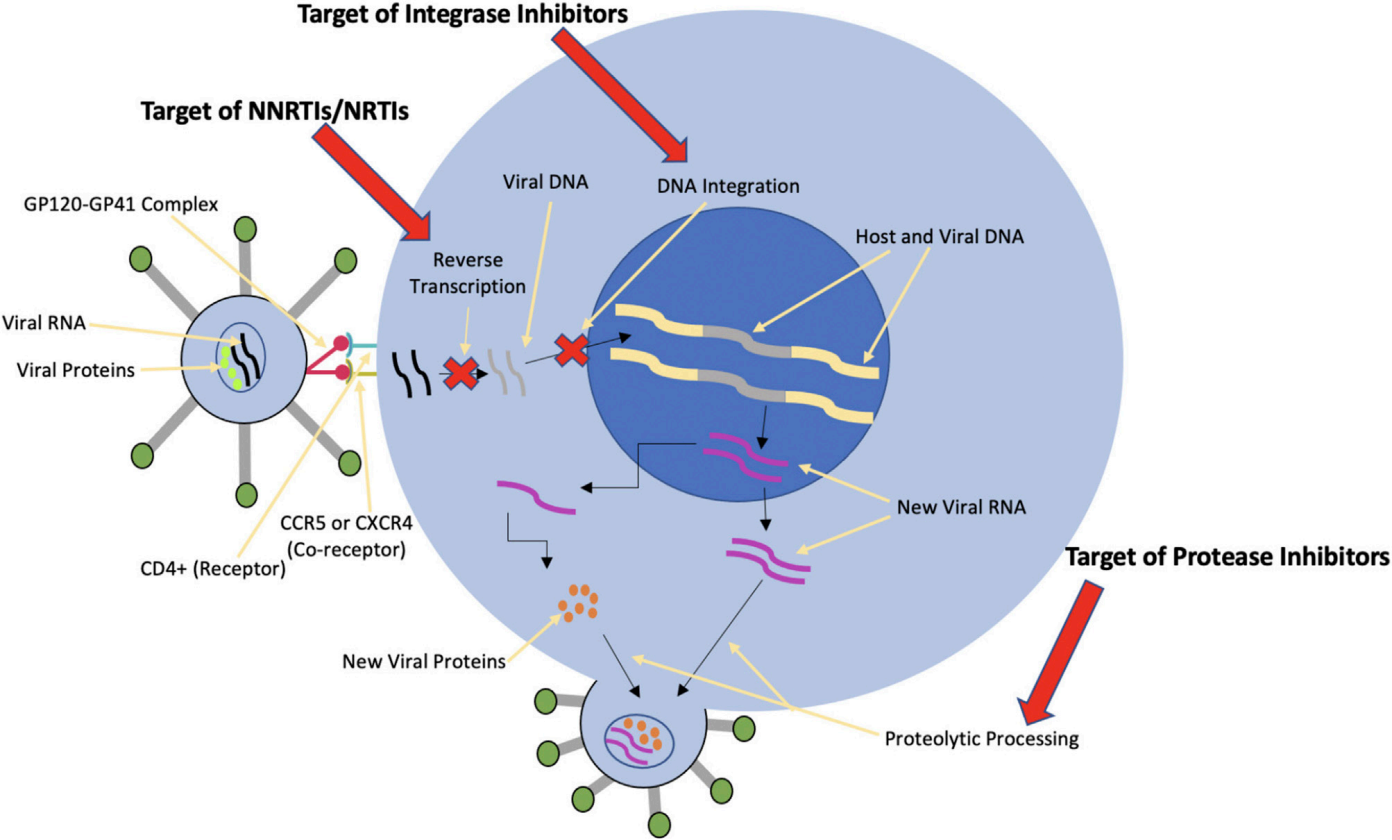
Combined Graphic of action of all cART drug classes.

Different classes of drugs each have their own unique effects on cardiovascular health. As the review of these classes and their associated effects draws to an end, the review will shift to highlighting specific therapeutic combinations of cART currently available (Figure 2). Of the several medications reviewed, newer combinations of cART such as Juluca (DTG/RPV), Biktarvy (BIC/FTC/TAF), and Complera (RPV/FTC/TDF) will be highlighted. Literature suggests an overall beneficial effect on cardiovascular health with bettered fasting triglyceride levels and lipid profiles. In contrast, Odefsey (RPV/FTC/TAF) presents with heightening cholesterol levels and worsened lipid profiles. Faced with these indications, it is imperative that clinicians weigh the cardiovascular risks and reward of cART regimes. Additionally, both the short and long-term effects of HIV infection and its treatments on parasympathetic and sympathetic function will be explored. Though still a relatively nascent field of study, the neurologic implications of HIV and its treatment therapies may prove to be significant. The intent of this paper is to present these regimes and provide an overview of medication mechanisms of action, protocol of use, and the cardiovascular and neurological effects.

### Viral proteins role in cardiovascular disease development

Before talking about the different classes of medications and their associations with cardiovascular disease progression. It is important to note that the HIV viral proteins themselves also have significant cardiovascular effects. The viral proteins Tat, Nef, and Env have been shown to be key factors in chronic inflammation that ultimately leads to endothelial cell dysfunction and atherosclerosis (Perkins et al., 2023). Tat, in particular, is a multifunctional protein that can lead to the activation of transcription factors including NFkB (nuclear factor kB), TNFα/β, IL-2, and IL-6 (Hofman et al., 1993; Buonaguro et al., 1994; Perkins et al., 2023). This protein can also act like CC chemokines and interact with CCR2/3 to recruit monocytes that contribute to chronic inflammation. In addition, Tat will act at the level of endothelial cells by inducing expression of adhesion receptors ICAM-1, VCAM-1 and E-selectins (Hofman et al., 1993; Perkins et al., 2023). These actions caused by Tat raise concerns for the development of cardiovascular sequelae in patients living with HIV. Another important protein to consider is Nef, a small accessory protein of HIV. This protein downregulates Major Histocompatibility Complex class I/II (MHC I/II) as well as CD80 and CD86 (Perkins et al., 2023). Nef protein will cause apoptosis in bystander T cells regardless of uninfected/infected state (Lenassi et al., 2010). Furthermore, this unique HIV accessory protein will lead to endothelial apoptosis and cholesterol dysfunction through the following mechanisms: First, Nef causes endothelial apoptosis and dysfunction *via* increased reactive oxygen species generation and decreasing endothelial nitric oxide synthase (eNOS) (Duffy et al., 2009). It has also been suggested that this protein leads to cholesterol metabolism dysfunction and lipid raft function which has implications on the development of atherosclerosis and cardiovascular disease (Bowman et al., 2019; Sviridov et al., 2020). Finally, the Env protein which in normal functioning of the HIV virus acts as the viral envelope protein. This HIV protein has been shown to induce expression ICAM-1, apoptosis of endothelial cells, and reduction of eNOS (Ullrich et al., 2000; Huang et al., 2001; Ren et al., 2002; Perkins et al., 2023). Additionally, Env proteins have been implicated in T-cell activation-dependent apoptosis and lead to antibody dependent cellular cytotoxic mediated killing of uninfected cells expressing CD4 (Banda et al., 1992; Forthal and Finzi, 2018). Altogether, these studies have shown links between these key HIV viral proteins Tat, Env and Nef and the roles they play on chronic inflammation and apoptosis. These findings have further implications on the role the virus itself plays in causing cardiovascular and neurologic sequelae.

### Protease inhibitors

Protease inhibitors (PI) are a widely used class of drugs in the treatment of HIV. Their therapeutic mode of action is to selectively bind to viral proteases, specifically HIV aspartyl protease, blocking proteolytic cleavage of protein precursors that are needed to produce viral particles. The main goal with PI therapy is to reduce the viral load of the HIV virus in the body to undetectable levels. This inhibits the HIV virus’s ability to replicate itself and reduces the viral load which slows the virus’s progression and helps to alleviate the symptoms for patients undergoing PI therapy. Protease inhibitors as a form of antiretroviral therapy is important and effective for PLHIV, however, there has been evidence in the literature that PI therapy can have effects on the patient’s cardiovascular system outside of its therapeutic uses (Antony et al., 2020). Specifically, this portion of the review will focus on antiretroviral protease inhibitors and their effects on the cardiovascular system.

There is extensive literature showing that protease inhibitors have an overall negative effect on the cardiovascular system (Mercié et al., 2002; Seminari et al., 2002; Zhou et al., 2005; Friis-Møller et al., 2007; Antony et al., 2020). In particular, PLHIV on PI therapy have exhibited increases in both total cholesterol and triglyceride levels (Friis-Møller et al., 2010), increased risk for atherosclerosis (Mercié et al., 2002; Seminari et al., 2002; Zhou et al., 2005) and increased risk for myocardial infarction (Friis-Møller et al., 2007). Sankatsing et al. (Sankatsing et al., 2009; Friis-Møller et al., 2010) performed an observational study of 11 previously established cohorts of HIV-infected patients looking at known coronary heart disease (CHD) risk factors including laboratory makers such as triglycerides and total cholesterol (LDL, HDL, VLDL). They found that PLHIV taking single or dual PI therapy were more likely to develop dyslipidemia with respect to both triglyceride as well as total cholesterol levels. Furthermore, their results showed that PLHIV who were on a dual PI-regimen had a higher risk of dyslipidemia compared to patients that were on a single PI-regimen. When comparing between classes of antiretroviral drugs, PLHIV on a single PI-regimen had a higher risk of dyslipidemia in comparison to PLHIV on non-nucleoside reverse transcriptase inhibitors (NNRTI) regimens. Reiss et al. (Worm et al., 2010) acknowledges that while their study shows a clear correlation between antiretroviral therapy and changes in lipid profiles, the exact mechanism to explain changes in plasma lipid levels and risk of CHD for PLHIV has not been established and requires further research.

While the exact mechanism linking lipid level changes and risk for CHD in PLHIV undergoing protease inhibitor therapy remains unclear, it is understood that increased total cholesterol and triglyceride levels lead to thickening of the arterial wall causing atherosclerosis. It has been reported that patients on protease inhibitor regimens have more pronounced atherosclerosis when compared to patients not undergoing HIV treatment (Mercié et al., 2002; Seminari et al., 2002). A prospective study performed by Mercie et al. (Mercié et al., 2002) measured the arterial intima media thickness (IMT) using high resolution B-mode ultrasonography. Their study revealed that PLHIV on cART with a PI had a larger IMT in comparison to patients on cART not with a PI. These findings were further corroborated by a similar study performed by Seminari et al. (Seminari et al., 2002) in which they found that PI-treated PLHIV had significantly larger and thickened IMT when compared to HIV-negative patient controls. The mechanism to explain the progression of atherosclerosis *via* PI therapy was noted by Zhou et al. (Zhou et al., 2005) in which they indicate that PI’s ability to activate the Unfolded Protein Response (UPR) may play an important role. In this process, UPR induces apoptosis in macrophages which are known to be involved in atherosclerotic lesions and atherosclerosis progression. However, this could be a dose and drug dependent process as they note that different PI’s activate the UPR at varying concentrations/dosages. In particular, their study indicates UPR activation of ritonavir, indinavir, and nelfinavir was estimated to be 5–15 μm, 15μm, 1–10 μm, respectively. They concluded that there is a significant relationship between PLHIV on PIs, atherosclerosis, and cardiovascular disease, but they could not exclude the involvement of other proteins as PIs can have different effects depending on the cell type. These findings regarding PI induced atherosclerosis have led to investigation of the incidence of myocardial infarction and antiretroviral therapy.

A study performed by the DAD study group followed a large group of 94,469 PLHIV looking at the incidence of myocardial infarction (MI) (Friis-Møller et al., 2007). The results of their study indicated that 345 PLHIV in their cohort had a MI, 90.4% of which had been exposed to PI therapy. Additionally, their research showed that patients taking protease inhibitors not only have an increased risk/rate of MI, but this risk is even higher for PLHIV that have had prolonged exposure to PI. The incidence of MI rose from 1.53 per 1,000 person-years in PLHIV not exposed to PI to an incidence of 6.01 per 1,000 person-years in PLHIV on PI for more than 6 years. The study demonstrates that there is a significant association between PI therapy and MI, but the mechanism by which this occurs remains to be discovered. Further investigation is required to understand the link between PI induced changes in lipid profiles, atherosclerosis, and incidence of MI.

### Integrase strand transfer inhibitors

Integrase Strand Transfer Inhibitors (INSTI) comprise a class of antiretroviral drugs that work by blocking the action of the HIV integrase enzyme. HIV’s integrase is important for the retrovirus to insert its own genetic information into the CD4 cells DNA and utilize the cell’s machinery to replicate itself. INSTIs beneficial pharmacological effects come from preventing this process from occurring. Similar to protease inhibitors it is helpful in keeping the viral load of the PLHIV low to undetectable levels. It is well understood that PLHIV are immunosuppressed, and at risk of numerous complications. Current treatment options such as INSTIs do not cure the patients of the retrovirus but have been successful in managing the disease. The purpose of this portion of the review will seek to understand the relationship between antiretroviral INSTI therapy and the heart/cardiovascular system.

Current research on integrase inhibitors and their effects on the cardiovascular system is limited. Nevertheless, the available research describes that INSTIs present a lower risk of cardiovascular disease (CVD) (Antony et al., 2020; O'Halloran et al., 2020; Fichtenbaum, 2010). A recent retrospective study done by O’Halloran et al. (O'Halloran et al., 2020) looked at major adverse cardiac events (MACE) including ischemic strokes, acute MI, and coronary artery bypass graft (CABG) over the course of 7 years (2008–2015). Their research demonstrated that PLHIV on an INSTI therapy exhibited about a 26% lower risk of significant MACE when compared to PLHIV that were not using any integrase inhibitors. Additionally, they reported that PLHIV with one or more CVD risk factors were switched to INSTI therapy. During the time period of 2008–2012, 51.6% of PLHIV on INSTI had at least 1 CVD risk factor compared to 45% PLHIV on other classes of antiretrovirals. Between 2013–2015, these percentages changed significantly to 50.1% and 51.1%, respectively. This trend was attributed to the fact that there was a significant movement of patients towards integrase inhibitor-based regimens because they had less harmful side effects and led to a decrease in CVD related risk in PLHIV. In their unadjusted model, O’Halloran et al. (O'Halloran et al., 2020) showed that there was no significant effect of INSTI therapy on CVD risk which they attributed to a higher incidence of MACE in patient population with more CVD risk factors. When adjusted for propensity score, they found that INSTI based regimens exhibited an overall decrease in risk of MACE.

The literature has clearly shown that INSTIs have been associated with low risk of CVD, but there has not been any relationship between INSTIs and dyslipidemia noted. On the other hand, there have been a few studies that have suggested that INSTIs may lead to significant increase in weight compared to PLHIV on other antiretroviral therapies (Norwood et al., 2017; Venter et al., 2019; Bourgi et al., 2020). Indeed, obesity and weight gain are widely recognized risk factors of cardiovascular disease, however, it is still not clear if the integrase inhibitor drugs induced weight gain is an important predictor for CVD.

### Nucleoside reverse transcriptase inhibitors

Nucleoside Reverse Transcriptase Inhibitors (NRTI) are yet another option of antiviral treatment therapy for persons living with Human Immunodeficiency Virus (HIV). NRTIs are usually prodrugs that are activated by tyrosine kinase enzymes in the host’s cells after administration. NRTIs lack a 3′-hydroxyl group that prevents the formation of a 3′-5′-phosphodiester bond in growing DNA chains and can disrupt replication of the HIV virus. Most notably, their incorporation during RNA/DNA-dependent DNA synthesis inhibits the production of both positive and negative strands of the DNA (Patel and Zulfiqar, 2022). NRTIs have long been established as a reliable and efficacious form of HIV antiviral therapy. However, there has been much speculation on whether they cause adverse effects on the patient’s cardiovascular system.

Many studies have found an association between the exposure/use of NRTIs and adverse effects on the heart and cardiovascular system, at large. Antony et al. (2020) (Antony et al., 2020) suggested that NRTIs such as abacavir, zidovudine, didanosine have generally been correlated with altered lipid metabolism and stavudine has been linked with increases in cholesterol, LDL cholesterol, HDL cholesterol, and triglycerides. The lipid elevating aspect of NRTIs has been well documented in current literature; however, direct association of NRTI use and more specific forms of cardiovascular risk such as myocardial infarction and cardiovascular disease (CVD) has since been debated. Many studies have noted a strong association between the use of NRTIs during HIV antiviral therapy, especially abacavir, and the risk of the patient experiencing a myocardial infarction while receiving the drug regimen (Obel et al., 2010; Worm et al., 2010; Therapy/INSIGHT SfMoA-R DAD Study Groups, 2008; Lang et al., 2010; Eyawo et al., 2019; Durand et al., 2011; Bavinger et al., 2013). Additionally, Islam et al. (2012) (Islam et al., 2012) suggests that the use of NRTI based antiviral therapy in PLHIV is associated with an increased risk of developing cardiovascular diseases such as myocardial infarction (MI), ischemic heart disease (IHD), cardiovascular and cerebrovascular disease and coronary heart disease (CHD).

As previously mentioned, the NRTI drug abacavir has been particularly highlighted as a drug of interest in observing cardiovascular disease in HIV patients receiving antiviral therapy. However, there have been many other studies that refute the association between NRTI exposure/use and risk of developing CVD. These studies that include various techniques of case controls, meta-analyses, and systematic reviews indicated no such association of NRTI use, especially abacavir, with the development of cardiovascular disease (Brothers et al., 2009; Bedimo et al., 2011; Cruciani et al., 2011; Ribaudo et al., 2011; Ding et al., 2012). This is indeed intriguing as these studies directly conflict the results of many other well-established studies. This discrepancy in conclusion of this association warrants further research into the mechanisms of the effects that NRTI drugs have on the cardiovascular system. This is much needed as all these studies have focused solely on evaluating the relationship between CVD risk and NRTI use instead of the unknown pathogenesis that these drugs may use to exert residual cardiovascular effects.

### Non-nucleoside reverse transcriptase inhibitors

Non-Nucleoside Reverse Transcriptase Inhibitors (NNRTI) are also used as a form of antiviral treatment therapy for PLHIV. NNRTIs exert their effects by binding to the reverse transcriptase and creating a hydrophobic pocket next to the active site. This creates a new spatial configuration of the substrate-binding site to reduce the overall polymerase activity, hence, slowing down DNA synthesis (Patel and Zulfiqar, 2022). Introduced to the world in 1996, NNRTIs have also been used heavily as a stand-alone medication or in combined anti-retroviral therapy for treatment of HIV-1 (Sluis-Cremer, 2018). However, not much is known about the effect that NNRTI-based therapy has on the cardiovascular system of PLHIV.

Some studies suggest that there may be a positive effect of the use of NNRTIs on cardiovascular health (Fisac et al., 2003; Maggi et al., 2011). It was observed that nevirapine, a model NNRTI drug, was shown to significantly decrease total cholesterol, low-density lipoprotein cholesterol (LDLc) and triglycerides, while also increasing high-density lipoprotein cholesterol, all indicators of improving overall cardiovascular health. Overall, PLHIV treated with nevirapine were seen to have a better lipid and glucose profile along with a lower tendency to develop subclinical atherosclerotic lesions (Maggi et al., 2011).

Additionally, many studies have also found no such association between the exposure/use of NNRTIs and adverse effects on the heart and cardiovascular system, at large (Friis-Møller et al., 2007; Lang et al., 2010; Worm et al., 2010; Islam et al., 2012; Silverberg et al., 2014; Rosenblatt et al., 2016; Eyawo et al., 2019). Most notably, after conducting multiple risk analyses, Eyawo et al. (2019) (Eyawo et al., 2019) suggested that their overall relative risk calculations (RR: 1.02; 95% CI 0.97–1.08) which indicates that total NNRTI exposure as a class, or as individual drugs (nevirapine and efavirenz), is not significantly associated with an increased risk of experiencing any myocardial infarction events per each year of exposure. Additionally, Lang et al. (2010) (Lang et al., 2010) concluded in their study that there was not an association found between the risk of experiencing a myocardial infarction and exposure to model NNRTIs such as efavirenz (OR, 1.01) or nevirapine (OR, 1.00).

As previously mentioned, the evidence supporting the hypothesis that there is no observed relationship between NNRTI exposure/use and cardiovascular health risk or even possible positive effects of these medications on cardiovascular health is plentiful. However, there are a few studies that have encountered mixed results or even a possible association between NNRTI exposure/use and cardiovascular health risk. Most notably, Tripathi et al. (2014) (Tripathi et al., 2014) suggested that the length of exposure to both PIs and NNRTIs was associated with a higher risk of developing cardiovascular disease. However, because NRTIs are usually prescribed along with PI or NNRTI regimens, they proposed that this association is at least partly caused by NRTI drug exposure. Likewise, other studies (Jamaluddin et al., 2010; Durand et al., 2011) noted adverse effects of NNRTI use on the cardiovascular system. Jamaluddin et al. (2010) (Jamaluddin et al., 2010) suggested that efavirenz, another NNRTI drug, increases endothelial cell permeability in human coronary arteries which may be due to the decrease of tight junction proteins and the increase of superoxide anion. Additionally, Durand et al. (2011) (Durand et al., 2011) discovered an increased risk of acute myocardial infarction with any exposure to the NNRTI drug efavirenz and delavirdine. These drugs have not previously been associated with adverse cardiac outcomes. This is indeed interesting as these studies directly conflict the results of many other well-established studies. This discrepancy would also warrant further research into the mechanisms of the effects that NNRTI drugs have on the cardiovascular system. This is much needed as all these studies have focused solely on evaluating the relationship between CVD risk and NNRTI use instead of the unknown pathogenesis that these drugs may use to exert residual cardiovascular effects.

### Combination anti-retroviral therapy

Combination anti-retroviral therapy also was known as highly active antiretroviral therapy (HAART), is a broad category pharmaceutical regimen for the management and treatment of HIV-1. Combination anti-retroviral therapy involves the co-administration of three or more drugs to target HIV-1 *via* different mechanisms, primarily to reduce plasma viral load and limit transmission (Shafer and Vuitton, 1999). Co-administration of antiretroviral drugs aims to inhibit replication of HIV in a multi-targeted approach. Acquired Resistance to one medication does not reduce the effectiveness of the treatment regimen. The goals of this treatment protocol are also multi-faceted. It aims to reduce morbidity and mortality rates from AIDS- and non-AIDS-related causes, improve patient quality of life, mitigate viral RNA load, prevent transmission, improve immunity, and avoid drug resistance (Eggleton and Nagalli, 2022).

As aforementioned, combined drugs in cART aim to inhibit the viral life cycle at various target stages. Among the primary six classes of cART agents, NRTIs, NNRTIs, PIs, INSTIs, FIs, and CCR5 antagonists, 25 different medications are available to mix and match (Table 2). Nucleoside Reverse Transcriptase Inhibitors (NRTIs) such as abacavir, didanosine, lamivudine, stavudine, tenofovir, and zidovudine, inhibit viral replication by competitively inhibiting reverse transcriptase. Non-nucleoside Reverse Transcriptase Inhibitors (NNRTIs), such as delavirdine, efavirenz, nevirapine, and rilpivirine, allosterically inhibit HIV reverse transcriptase and thereby inhibit DNA polymerase. Protease inhibitors (PIs), such as atazanavir, darunavir, indinavir, work to produce non-infectious virions from HIV-infected cells *via* proteolysis of gag/pol polyproteins. These inhibitors are often reserved for patients who have not responded well to cART and require boosting agents (ritonavir or cobicistat). Integrase Strand Transfer Inhibitors (INSTIs), such as dolutegravir, elvitegravir, raltegravir prevent viral DNA from being integrated into the host genome by binding to viral integrase. Fusion inhibitors (FIs), particularly enfuvirtide, bind glycoprotein gp41 and provide the virus immunity against CD4 T-cells. Chemokine Receptor Antagonists (CCR5 Antagonists), including maraviroc, reversibly block the gp120-CD4 interaction to prevent viral uptake by the CD4 T-cell. Typically, cART involves two NRTIs alongside one of either PI, NNRTI, or INSTI, the latter preferred over the former two (Antony et al., 2020).

**TABLE 2:** cART Drug Combinations and Cardiovascular Health Risk.

Drug	Cardiovascular Effects	References
*Biktarvy (BIC/FTC/TAF)*	• Improved lipid profiles	Deeks et al., 2018 Whol et al., 2018
*Complera (RPV/FTC/TDF)*	• Improve lipid profiles and reduced formation of atherosclerotic lipid plaques • No significant change in CV risk	Tungsiripat et al., 2010 Plum et al., 2021
*Odefsey (RPV/FTC/TAF)*	• Elevated lipid levels, dyslipidemia and pro-atherogenic lipoproteins • Significant increase in CV risk	Lacey et al., 2021 Cid-Silva et al., 2019 Plum et al., 2021
Juluca (DTG/RPV)	• Improved lipid profiles • Neutral effect on lipid profile	Palacios et al., 2018 Ciccullo et al., 2019 Ribera et al., 2019 Capetti et al., 2018 Dowers et al., 2018

Green = Beneficial Effects, Red = Harmful Effects, Gray = No Significant change/Neutral.

The administration of these classes of medications varies based on the patient and physician factors. Virological and immunological benefits of initiating treatment early on must be balanced with therapy costs, adverse effects, and the risk of resistant therapy (Shafer and Vuitton, 1999). Other factors include but are not limited to patient compliance and adherence, renal or hepatic impairment, drug resistance, pregnancy, or comorbidities such as hepatitis B, tuberculosis, or cardiovascular disease (Eggleton and Nagalli, 2022). The European AIDS Clinical Society (EACS) recommends cART in all HIV patients, regardless of CD4 counts. Immediate treatment is indicated for HIV patients with CD4 counts below 350 cells/μL, age over 50 years, pregnancy, severe symptoms, infections, and neurologic comorbidities (Antony et al., 2020). Even with these guidelines, past clinical studies show cART predisposes patients to adverse cardiologic effects.

A growing concern in recent years is about cART patients presenting an increased rate of cardiovascular diseases. At the cellular level, mitochondrial toxicity induced by cART is responsible for increased cardiac apoptosis, reduced repair mechanisms, block hyperplasia/hypertrophy, and decreased ATP production in the heart tissue (Dubé et al., 2008). A 2012 schematic review estimated CVD risk among PLHIV and found that those treated cART, particularly abacavir, are at a significantly increased risk of coronary vascular disease (Islam et al., 2012). Metabolic complications of cART include impaired glucose metabolism, lipodystrophy, visceral fat accumulation, subcutaneous fat loss, and dyslipidemia. Increased total cholesterol, low-density lipoproteins, and triglycerides in these PLHIV are associated with atherosclerosis, ischemic heart disease, and MI (Neto et al., 2013). This abnormal fat deposition in cardiomyocytes and lipodystrophy is linked with increased risk of cardiovascular disease and insulin resistance, predisposing cART receiving patients to adult-onset of type 2 diabetes (Pinto and da Silva, 2018). In the study by Neto et al. (2013) (Neto et al., 2013), it was found that cART is also linked to causing an increased thickness of the tunica intima-media complex, which may induce endothelial dysfunction and arterial stiffness. In a prospective observational study (Friis-Møller et al., 2007) it was found that the rate of MI increased by 26% within four to 6 years of receiving cART. Risk factors associated with MI in PLHIV included, but were not limited to, current/former smoking, previous CVD, male sex, high serum cholesterol, high triglyceride levels, and diabetes. The absolute risk of MI among the large cohorts of PLHIV remained low, suggesting such a risk must be balanced with the benefits of receiving cART for HIV. Another cohort study published in 2014 indicated an increased rate of MI among PLHIV receiving abacavir over tenofovir in their cART regimen (Brouwer et al., 2014). Given the small patient size in this study, a larger clinical trial was suggested to define this association. However, the observations of increased MI risk are fit to include in this review (Brouwer et al., 2014).

Combination anti-retroviral therapy is used as prophylactic therapy during pregnancy to prevent *in-utero* vertical transmission of HIV. First-trimester exposure to efavirenz, an NNRTI and teratogen, is associated with an increased risk of infant congenital abnormalities. Among the 80 anomalies including muscular, cardiovascular, renal, genitourinary, craniofacial, and nervous systems, the cardiovascular anomalies occurred most frequently (Knapp et al., 2012). Another prospective cohort study by Guerra et al. (2016) (Guerra et al., 2016) concluded that *in-utero* cART exposure is associated with reduced left ventricular mass, dimension, septal-wall thickness, and increased left ventricular fractional shortening and contractility during the first 2 years of life with pronounced effects in girls. With over 100,000 US infants exposed to cART, this suggests that long-term monitoring is essential for this HIV-negative population. Newborns positive for HIV present with progressive, chronic abnormalities in left ventricular structure and function. With that said, HIV-negative newborns exposed to cART may still show reduced left ventricular contractility at 5 years of age (Lipshultz et al., 2002; Lipshultz et al., 2011). Among children, those receiving cART present with smaller heart chambers, decreased left ventricular mass, lower heart rate, and increased left ventricular contractility, as compared to those unexposed to cART (Lipshultz et al., 2012). With this degree of knowledge, it is essential that children exposed to cART *in-utero* be monitored long-term regardless of HIV status to reduce the risk of cardiac mortality (Antony et al., 2020). Physicians are obligated to weigh the benefits of cART and associated cardiovascular risks. Cardiac screening and active monitoring are essential in those being evaluated for and receiving this combinatorial treatment regime. As PLHIV tend to live a longer life with HIV treatments, it is necessary to pay closer attention to those with pre-existing cardiovascular conditions, which are at risk of worsening with exposure to long-term cART. A better understanding of the molecular interactions and mechanisms involved in cART with HIV will further the scientific grasp of this concept (Neto et al., 2013).

Next in this review, we focus on delving into the multiple drugs used for antiretroviral treatment for PLHIV. The sections outline individual drug classes and a focus on cART is placed once a generalized review of individual classes is complete.

### Cellular effects of cART on CVD development

When discussing cART it is also important to understand the cellular implications and effects treatment has on PLHIV. The exact mechanisms by which cART leads to heart dysfunction and toxicity at the cellular level have not been elucidated. However, studies have been able to show that cART treatment leads to oxidative stress, cellular hypertrophy, and modified histones in cardiomyocytes (Kashyap et al., 2021a; Kashyap et al., 2021b). In addition, RNA-seq data analysis has demonstrated that differences in gene expression causes changes at the transcriptional level ultimately causing cardiac hypertrophy and toxicity (Kashyap et al., 2021a). In particular, they found through their studies of qRT-PCR of cART treated cardiomyocytes major genes associated with cardiac hypertrophy. The main genes that were found to be elevated in these cells and cardiac tissue of PLHIV treated with cART were Hopx, P2rx4, and Ackr3 (Kashyap et al., 2021a). The Hopx gene has been closely studied and found to be upregulated in cART-treated PLHIV and plays a key role with regards to cardiomyocyte hypertrophy. Kashyap et al. (2021) (Kashyap et al., 2021a) has also shown in their studies with knockout mice that Hopx expression causes increased size in cardiomyocytes while knockout mice showed size reduction. Furthermore, the Hopx gene has been proposed to negatively impact proliferation and diferentiation of cardiomyocytes (Chen et al., 2002). Overall, these findings on the molecular role that Hopx plays in the development of cardiac sequelae in PLHIV treated with cART cannot be ignored. Further exploration and study of these genes that act at the cellular level will help elucidate the mechanisms by which cardiac sequelae develops in cART treated HIV patients.

### Biktarvy

This section review aims to highlight the cardiovascular-related effects of combined anti-retroviral treatment (cART) for PLHIV with or without pre-existing heart comorbidities. cART has revolutionized the treatment with which PLHIV are treated. The tolerability, convenience, and potency of this treatment construct have made this standard for viral suppression. As discussed further in this review, cART combines three antiviral agents from at least two varying classes with different mechanisms of action. Treatment regimens customarily include two nucleoside reverse transcriptase inhibitors (NRTIs) and either an integrase strand transfer inhibitor (INSTI), non-nucleoside reverse transcriptase inhibitor (NNRTI), or a protease inhibitor (PI). Most often, the preferred of the three is an INSTI alongside the two NRTIs (Deeks, 2018). For some time, raltegravir, elvitegravir, and dolutegravir have been used in single-tablet regimens (STRs) with the two NRTIs (i.e., abacavir/lamivudine/dolutegravir). STRs with elvitegravir are often combined with boosting agents such as cobicistat (i.e., elvitegravir/cobicistat/emtricitabine/tenofovir alafenamide). Drug-drug interactions with this specific STR increased hypersensitivity to CVD risk, renal toxicity, bone toxicity (Deeks, 2018).

Bictegravir/emtricitabine/tenofovir alafenamide (BIC/FTC/TAF) or Biktarvy is a relatively new cART that was approved for the treatment of HIV/AIDS in February 2018. It is a fixed-dose combination drug that is taken once daily that contains bictegravir (integrase inhibitor), emtricitabine (NRTI), and tenofovir alafenamide (NRTI) at 50 mg, 200 mg, 25 mg, respectively. The current understanding of this STR regimen’s effects on the cardiovascular system is still in its early stages; however, it has been noted to have particularly significant effects on the patient’s triglyceride profile (Daar et al., 2018; Stellbrink and Hoffmann, 2018; Wohl et al., 2018). Daar et al. (2018) (Daar et al., 2018) reported that PLHIV taking Biktarvy had considerable improvement on fasting triglycerides as well as total-to-HDL cholesterol when compared to a boosted protease inhibitor group. Furthermore, Biktarvy showed similar lipid profiles to dolutegravir based therapies over a course treatment of 48 weeks (Deeks, 2018). This same trend was observed when looking at 96 weeks of treatment study by Stellbrink et al. (2018) (Stellbrink and Hoffmann, 2018); however, one study performed by Wohl et al. (2018) (Wohl et al., 2018) did show that lipid profiles were statistically significantly different for Biktarvy treatment regimens vs dolutegravir based therapies, but were deemed not clinically relevant (−0.1 vs −0.2 total cholesterol to HDL, +15 vs. +8 mg/dL total cholesterol, +17 mg/dL vs +7 mg/dL LDL). Given these lipid profile changes, implications on cardiovascular health on Biktarvy prescribed PLHIV should be considered. These include the propensity of atherosclerosis, as well as incidence/prevalence of other related adverse cardiovascular events. Apart from the mild increases in LDL-C, no other cardiovascular related effects are noteworthy.

### Juluca

This section of the review looks to explore Juluca, and its effects on cardiovascular effects. This combination therapy consists of a single tablet regimen that includes 2 HIV medications, the HIV integrase strand transfer inhibitor (INSTI), dolutegravir, and the non-nucleoside reverse transcriptase inhibitor (NNRTI), rilpivirine. As a combination drug, it utilizes both the mechanism of an INSTI, which is by blocking the action of HIV’s integrase is which inserts its own genetic information into the CD4 cells DNA and replicates using the cell’s machinery, and the mechanism of a NNRTI, which is by binding to the reverse transcriptase to reduce the overall polymerase activity, hence, slowing down DNA synthesis (Patel and Zulfiqar, 2022). Despite this, not much is known about the long-term cardiovascular effects that Juluca or similar combinations of drugs have on PLHIV.

With Juluca being a relatively newer drug regimen on the market, there is not a dearth of evidence that highlights the positive effects that it or identical drug combinations have on cardiovascular health. However, Palacios et al. (2018) (Palacios et al., 2018) found that switching from any antiretroviral regimen to a drug regimen of a tablet containing dolutegravir and rilpivirine (commonly known as DTG + RPV) lead to an improvement in lipid profile with a decrease of 12.7% in triglyceride levels, particularly in those previously on protease inhibitors only. Notably, total cholesterol, HDL and LDL levels remain unchanged. Additionally, Ciccullo et al. (2019) (Ciccullo et al., 2019) also noted that there was a noticeable decrease in total cholesterol levels in PLHIV that switched to a drug regimen containing dolutegravir and rilpivirine, with no other adverse effects on cardiovascular health. Ribera et al. (2019) (Ribera, 2018) and Capetti et al. (2018) (Capetti et al., 2018a; Capetti et al., 2018b) also cited a positive cardiovascular effect of the DTG + RPV combination with a decrease in the LDL-C/HDL-C ratio. However, there are some studies that suggest that this drug combination provides neutral effects/no change in cardiovascular health with its introduction. For example, in a literature review, Dowers et al. (2018) (Dowers et al., 2018), cited widely neutral effects on PLHIV lipid profile from the use of this drug combination. With this, they also noted that data is very limited and long-term studies are desperately needed to elicit more information about the effects that DTG + RPV has on cardiovascular health.

While there is some research noting both positive and neutral cardiovascular effects of the dolutegravir and rilpivirine drug combination in PLHIV, there are no such studies that cite a negative cardiovascular impact that this drug combination exerts. This may be due to some mechanistic, synergistic activity that occurs in this drug combination that contributes to both the tremendous viral suppression while also sparing/improving overall cardiovascular health. More insight into these mechanisms would be beneficial in determining how this drug combination affects cardiovascular function.

### Odefsey vs. complera

This section will focus on the transition from tenofovir disoproxil (TDF) to tenofovir alafenamide (TAF) based therapies with emphasis on Complera (RPV/FTC/TDF) and Odefsey (RPV/FTC/TAF). These two drugs are compositionally very similar except for the tenofovir prodrug used in Complera (tenofovir disoproxil fumarate (TDF)) vs. Odefsey (tenofovir alafenamide (TAF)). Complera is a fixed oral dose cART that possesses emtricitabine (FTC) (NRTI) 200 mg, rilpivirine (RPV) (NNRTI) 27.5 mg, tenofovir disoproxil fumarate (NRTI) 245 mg. Similarly, Odefsey is primarily taken *via* oral administration. The tablets are a cART of emtricitabine (FTC) (NRTI) 200 mg, rilpivirine (RPV) (NNRTI) 25 mg, and tenofovir alafenamide (TAF) (NRTI) 28 mg. It may be presumed given the natural progression of cART that Odefsey is the successor of Complera. The tenofovir alafenamide prodrug of tenofovir was favorably introduced into combination therapy because of its ability to achieve elevated intracellular concentrations, but with lower plasma tenofovir concentrations than tenofovir disoproxil fumarate (Plum et al., 2021). This property alone would lessen the long-term toxicity burden placed on the PLHIV that are taking this cART. Therefore, TAF is currently recommended for cART in comparison to TDF-related regimens. This section of the literature review aims to investigate the effects of TDF vs. TAF based therapy on the cardiovascular system.

Current literature on the RPV/FTC/TAF and RPV/FTC/TDF and their respective cardiovascular effects is limited; however, what has been thoroughly explored is their relative effects on lipid profile and dyslipidemia. It has been shown that TDF can improve lipid profiles and reduce the formation of atherosclerotic lipid plaques (Tungsiripat et al., 2010). On the other hand, TAF based therapies have been shown to have unfavorable effects on lipid profiles (Cid-Silva et al., 2019; Lacey et al., 2020). Plum et al. (2021) (Plum et al., 2021) performed an observational retrospective study of PLHIV outpatients from CHU Liege hospital in Belgium in which they calculated cardiovascular risk and measured lipid profiles between two groups (TDF/TDF vs TDF/TAF) over a span of 2 years (2016–2018). They compared patients in a TDF/TDF group who were PLHIV that were treated continuously with TDF-based therapy against those in a TDF/TAF group that were PLHIV who were initially started on TDF based therapy for at least 6 months and switched to a TAF based therapy. Between 2016–2018, patients in the TDF/TDF group saw statistically significant decreases in total cholesterol levels (Plum et al., 2021). There was no significant change in the triglyceride/HDL ratio in the TDF/TDF group (Plum et al., 2021). PLHIV in the TDF/TAF group during that period saw statistically significant increases in total cholesterol levels, triglycerides, and HDL. There was also no significant change in the triglyceride/HDL ratio in this group (Plum et al., 2021). Plum et al. also calculated cardiovascular (CV) risk between the two groups using The Data collection on Adverse Effects of Anti-HIV Drugs Study (DAD) equation. Using this algorithm, they found significant differences in CV risk between the two groups. Those of the TDF/TDF group did not see a change in CV risk while those in the TDF/TAG group saw an increase in CV risk. The results from the study performed by Plum et al. (2021) (Plum et al., 2021) confirms that switching from TDF to TAF based cART worsens lipid profile and pro-atherogenic lipoproteins, a finding that has largely been demonstrated by many other studies (Gotham et al., 2017; Milinkovic et al., 2019; Lacey et al., 2020). In their study, they concluded that while TAF therapies can be enticing due to its lower serum concentrations and reduced toxicity; but there is significant concern regarding cardiovascular health and notable increases in proatherogenic lipids. It may be worth considering lipid lower medications in PLHIV who are taking or considered for TAF based cART therapies. Plum et al. acknowledges while the study does offer significant insight on potential CVD in the TDF to TAF switch, further studies and longer-term follow-up is needed to assess if cardiovascular event incidence is accurately anticipated by the reduced DAD algorithm.

### Neurological sequelae of cART

It has been reported that patients with HIV infection have frequent autonomic nervous system (ANS) impairments (Robinson-Papp et al., 2013). Before the widespread use of cART, studies conducted at the beginning of the AIDS epidemic revealed that autonomic dysfunction presented a significant neurologic consequence of HIV (Craddock et al., 1987; Cohen and Laudenslager, 1989; Villa et al., 1992; Villa et al., 1995). Even though the sample sizes for these investigations ranged from 5 to 57 PLHIV, drug use that might resemble autonomic phenomena was routinely ignored, and all but one ANS testing was done in isolation. Hence, it is difficult to determine if the abnormalities in the ANS were a component of a larger neuro-AIDS syndrome, a weakness in a single area of the nervous system, or even a side effect of complicating pharmaceutical use (Table 3) (Robinson-Papp et al., 2013).

**TABLE 3:** Summary of ANS effects of HIV and cART therapy.

References	ANS Effects/Consequences
**Chow et. al., 2012**	• No discernible effects on ANS. • No short term complications
**Gluck et. al., 2000**	• Autonomic and sensorimotor neuropathies
**Lebech et. al, 2007**	• Cardiovascular autonomic neuropathy • Higher resting HR. • Greater LF:HF ratio • Parasympathetic dysfunction
**Mittal et. al., 2004**	• Cardiovascular autonomic neuropathy
**McIntosh et. al, 2017**	• Low HRV and parasympathetic tone • Parasympathetic withdrawal
**Cole et. al, 2001**	• Neural activity promotes viral replication
**Fliers et. al, 2003**	• Body fat distributionviaautonomic balance shift → Adipose redistribution → Cardiovascular consequences

Red = Harmful Effects, Gray = No Significant change/Neutral.

Compared to the previously mentioned studies, early cART studies had similar findings and limitations (Rogstad et al., 1999; Neild et al., 2000). However, one study by Glück et al. (2000) (Glück et al., 2000) on 61 PLHIV revealed both autonomic and sensorimotor neuropathies without stating whether they were associated. Nonetheless, a few articles on cardiovascular autonomic neuropathy have shown mild or subclinical abnormalities (Mittal et al., 2004; Lebech et al., 2007) or symptoms without obvious deficits (Compostella et al., 2008). A study conducted on 97 PLHIV revealed that mild autonomic dysfunction was related to medical factors but not HIV immuno-virologic ones (Askgaard et al., 2011). This conclusion was later confirmed in a preliminary report by Chow et al. (2012) (Chow et al., 2012) investigating the effects of starting a new cART regimen on the autonomic function in PLHIV. Most of the naive participants began their cART treatment with tenofovir, emtricitabine, and efavirenz. This study demonstrated that when cART is started early in the course of the disease, there are no discernible variations in how it affects the ANS as determined by cardiovagal, adrenergic, and sudomotor tests. Hence, the short-term complications in autonomic function may not be caused by cART (Chow et al., 2012).

Since neurotransmitters can speed up the replication of HIV *in vitro*, it is possible that the variations in ANS could boost the replication in individuals receiving highly active cART. To elaborate, prior to receiving cART, PLHIV who displayed constitutively high levels of ANS activity had worse plasma viral load reduction and worse CD4^+^ T cell recovery throughout the course of 3–11 months of treatment. Norepinephrine however increased replication of both CCR5-and CXCR4-tropic strains of HIV *in vitro via* up-regulation of chemokine receptors and enhanced viral gene expression, suggesting that neural activity may directly promote residual viral replication. In PLHIV receiving cART, the current data show an unexpectedly high linear correlation between inherent individual differences in ANS activity and recurrent viral replication (Cole et al., 2001).

cART has made autonomic failure more widely recognized in PLHIV, but the impact of HIV on Heart Rate Variability (HRV) is less clear. In fact, a meta-analysis on HRV showed that PLHIV had lower HRV and parasympathetic tone along with reduction in both low-frequency (LF) and high-frequency (HF) domains, but a greater LF:HF ratio in the frequency domain. In other words, LF reduction was smaller than that of HF, which may indicate parasympathetic withdrawal (McIntosh et al., 2017). An overall decline in autonomic function with a shift toward sympathetic dominance was also observed in a relatively young group of PLHIV on cART. This change may put PLHIV at an earlier and higher risk of arrhythmias, cardiac events, and a quicker course of the virus. Accordingly, the inflammation-immune dysfunction could explain the cause of the reduced heart rate variability associated with HIV (McIntosh et al., 2017). However, Chow et al. (2011) (Chow et al., 2011) conducted a study which showed that both viremic and aviremic patients showed impaired parasympathetic function when compared to PLHIV. Even with the high occurrence frequency of autonomic dysfunction in PLHIV, physicians hardly notice it, and symptoms frequently go undiagnosed or are erroneously related to pharmaceutical side effects leading to higher risks of morbidity, including QTc prolongation and dangerous cardiac arrhythmias (Robinson-Papp et al., 2013).

In another study by Lebech et al. (2007) (Lebech et al., 2007), cART significantly altered the course of the illness, improved prognosis, and reduced morbidity. In addition, PLHIV resting heart rates were higher than those of controls. Both total HRV and parasympathetic activity were lower in the PLHIV than in the controls. When compared to the control group, low frequency power was lower in the PLHIV group. Furthermore, systolic and diastolic blood pressures, as well as high frequency power, were not different amongst the groups. As such, increased resting heart rate and decreased short-term heart rate variability in HIV patients on cART are signs of parasympathetic dysfunction (Lebech et al., 2007). On another note, Gupta et al. (2022) (Gupta et al., 2022) showed that patients on cART have greater levels of carotid intimal medial thickness and high-sensitivity C reactive protein than cART-naive participants which are major CVD risk factors.

In addition, there is no known explanation for the abnormal body fat distribution seen in HIV associated Adipose Redistribution Syndrome (HARS). White adipose tissue is regulated by the brain as well as humoral mechanisms. While parasympathetic innervation has an anabolic effect on white adipose tissue, sympathetic innervation promotes lipolysis. Results of neuroanatomical research revealed a distinct somatotopy regarding the sympathetic and parasympathetic branch’s control over white adipose tissue, with different groups of autonomic neurons innervating either the subcutaneous or the visceral fat compartment. The CNS is therefore a significant factor in controlling the distribution of body fat. Hence, HARS might be caused by the CNS side effects of antiretroviral therapy and may signal a shift in the autonomic balance that causes adipose tissue to be distributed differently leading to cardiovascular deleterious predisposition and consequences (Fliers et al., 2003).

## Limitations

As we read through this review, we need to consider certain limitations that pertain to many studies done and presented. For instance, the protocol/evidence-based standard for medication for HIV management is constantly being altered attempting to improve the health outcome of PLHIV. Therefore, best practice in terms of which combination of drugs are most effective, with minimal exposure to adverse outcomes, is changed frequently. This makes it difficult to longitudinally follow the effects of these drugs on PLHIV as healthcare providers constantly adjust their treatment regimen. This comes with more constraints when PLHIV are concomitantly treated for other concurrent or subsequent disease, such as the cardiovascular ones. Even variations within the population of PLHIV, based on social, economic, and genetic factors can derail the longitudinal analysis of the long-term effects of HIV antiretroviral therapy, if not accounted for in the analysis. With the changing therapies, it is important to note the lack of information regarding the interactions of these new HIV drugs with current medication regimens that are commonly used to treat cardiovascular diseases. This should be an area of research as advances toward treatment continue. On the other hand, PLHIV often presents with concomitant diseases and other risk factors that may confound the incidence of the development of cardiovascular treatment. Accordingly, it is well established that PLHIV have compromised immune systems, dyslipidemia, and atherosclerosis which sets them up for higher prevalence of cardiovascular comorbidities and worse health outcomes. This can adversely affect any correlation found between HIV and CVD. Furthermore, some data may be skewed due to errors in sampling from populations of differing socioeconomic status. Higher incidence of HIV is seen in areas of low socioeconomic status and impoverished areas. The limited access to healthcare and education leads to riskier health practices and higher rates of HIV infections as well as other diseases in these areas and populations, especially cardiovascular diseases, and related comorbidities. Thus, a careful interpretation of research outcomes is of essence while taking these variants into consideration.

## Conclusion

Human Immunodeficiency Virus is a well-known disease that continues to affect millions of people across the world. Since its discovery, therapy has rapidly evolved from single therapies to the well sought after cART. cART has been revolutionary to controlling viral load, but as treatments evolve and new combination drugs are made there begs the concern of the long-term consequences of these therapies. This article has extensively outlined the individual drug classes and their respective effects on the cardiovascular system. Overall, integrase inhibitor and NNRTI based therapies are more consistently correlated with lower risk of cardiovascular disease while protease inhibitors are strongly linked to increased risk of cardiovascular disease and ultimately worse outcomes. NRTI based therapies correlate with a negative cardiovascular impact, but these indications were based on and calculated by CVD risk models. This review also evaluated selective cART regimes as this has become the popular standard for treating PLHIV. Majority of the studies including Juluca (DTG/RPV), Biktarvy (BIC/FTC/TAF), Complera (RPV/FTC/TDF) suggest an overall positive effect on cardiovascular health that was extrapolated from the improvements on fasting triglyceride levels and lipid profiles. In contrast, cART such as Odefsey (RPV/FTC/TAF) had significant increases in cholesterol levels and worse lipid profiles in PLHIV.

The literature and data surrounding the lipid profiles of PLHIV that take cART therapies are vast. However, many of the studies only assess cardiovascular disease prediction models. While it is true many of these therapies are relatively new and thorough cardiovascular assessments may be limited, research on the cardiovascular repercussions is crucial to accurately evaluate patient risk. In addition, this would offer insight into the clinical significance of the changes in lipid profile that many papers have outlined in their studies in the development of debilitating disease.

There are also some other limitations that we encountered while conducting this review. For example, the protocol/evidence-based standard for medication for HIV management is constantly being altered, especially pertaining to those PLHIV, with concurrent or subsequent cardiovascular disease. Additionally, PLHIV often present with concomitant diseases and other risk factors that may confound the incidence of the development of cardiovascular treatment. This can adversely affect any correlation found between HIV and cardiovascular disease. Furthermore, some data may be skewed due to errors in sampling from populations of differing socioeconomic status. Hence, this can affect the results of the studies that were used in this review.

Finally, HIV antiretroviral therapy effects on the CNS, particularly the ANS, cannot be ignored. The literature indicates that PLHIV receiving cART had a strong correlation between ANS activity and viral replication with higher ANS activity indicating worse outcomes. PLHIV on cART show a clear sympathetic dominance and parasympathetic dysfunction which is concerning for the potential for adverse cardiac events as well as HARS. Taken together, the autonomic imbalance associated with HIV antiretroviral therapy must be accounted for prior to starting antiretroviral therapy as it can have strong implications on the prognosis of patients on/starting cART therapy.
