# Supplementary material for: Direct and indirect cardiovascular and cardiometabolic sequelae of the combined anti-retroviral therapy on people living with HIV
Source: Front Physiol. 2023 Mar 27;14:1118653. doi: 10.3389/fphys.2023.1118653 (PMC10107050; doi:10.3389/fphys.2023.1118653)
Supplement: Supplementary file 1 [file DataSheet2.PDF]

Drug	Cardiovascular Effects	References
Biktarvy (BIC/FTC/TAF)	• Improved lipid profiles.	Deeks et al., 2018<br>Whol et al., 2018
Complera (RPV/FTC/TDF)	• Improve lipid profiles and reduced formation of atherosclerotic lipid plaques.<br>• No significant change in CV risk.	Tungsiripat et al., 2010<br>Plum et al., 2021
Odefsey (RPV/FTC/TAF)	• Elevated lipid levels, dyslipidemia and pro-atherogenic lipoproteins.<br>• Significant increase in CV risk.	Lacey et al., 2021<br>Cid-Silva et al., 2019<br>Plum et al., 2021
Juluca (DTG/ RPV)	• Improved lipid profiles<br>• Neutral effect on lipid profile	Palacios et al., 2018<br>Ciccullo et al., 2019<br>Ribera et al., 2019<br>Capetti et al., 2018<br>Dowers et al., 2018
