# Supplementary material for: Direct and indirect cardiovascular and cardiometabolic sequelae of the combined anti-retroviral therapy on people living with HIV
Source: Front Physiol. 2023 Mar 27;14:1118653. doi: 10.3389/fphys.2023.1118653 (PMC10107050; doi:10.3389/fphys.2023.1118653)
Supplement: Supplementary file 2 [file DataSheet3.PDF]

Reference	ANS Effects/Consequences
**Chow et. al., 2012**	• No discernible effects on ANS.<br>• No short term complications.
**Gluck et. al., 2000**	• Autonomic and sensorimotor neuropathies.
**Lebech et. al, 2007**	• Cardiovascular autonomic neuropathy.<br>• Higher resting HR.<br>• Greater LF:HF ratio.<br>• Parasympathetic dysfunction.
**Mittal et. al., 2004**	• Cardiovascular autonomic neuropathy.
**McIntosh et. al, 2017**	• Low HRV and parasympathetic tone.<br>• Parasympathetic withdrawal.
**Cole et. al, 2001**	• Neural activity promotes viral replication.
**Fliers et. al, 2003**	• Body fat distribution via autonomic balance shift → Adipose redistribution → Cardiovascular consequences
