# Supplementary material for: Direct and indirect cardiovascular and cardiometabolic sequelae of the combined anti-retroviral therapy on people living with HIV
Source: Front Physiol. 2023 Mar 27;14:1118653. doi: 10.3389/fphys.2023.1118653 (PMC10107050; doi:10.3389/fphys.2023.1118653)
Supplement: Supplementary file 3 [file DataSheet1.PDF]

**Drug**	**Cardiovascular Effects**	**References**
Protease Inhibitors	• Increased cholesterol and triglyceride levels.<br>• Increased risk of atherosclerosis Increased risk myocardial infarction	Fontas et al., 2020;<br>Zhou et al., 2005;<br>Seminari et al., 2002;<br>Mercie et al,. 2002;<br>DAD Study Group, 2007.
Integrase Inhibitors	• Lower risk of cardiovascular disease and major cardiac events	O'Halloran et al., 2020;<br>Fichtenbaum et al., 2010;<br>Antony et al., 2020.
Nucleoside Reverse Transcriptase Inhibitors	• Elevated lipid levels, cholesterol, and triglycerides<br>• Increased risk of cardiovascular disease<br>• Increase risk myocardial infarction	Antony et al., 2020;<br>Islam et al., 2012;<br>DAD Study Group et al, 2008;<br>Lang et al., 2010;<br>Obel et al., 2010;<br>Ewayo et al., 2019;<br>Durand et al., 2011;<br>Worm et al., 2010;<br>Bavinger et al., 2013.
Non-Nucleoside Reverse Transcriptase Inhibitors	• Improved lipid and glucose profiles<br>• No significant association with cardiovascular risk<br>• Increased endothelial permeability.<br>• Increased risk myocardial infarction	Maggi et al., 2011;<br>Fisac et al., (2003);<br>DAD Study Group et al. 2007;<br>Worm et al., 2010;<br>Silverberg et al., 2014;<br>Islam et al., 2012;<br>Eyawo et al., 2012;<br>Lang et al., 2010;<br>Rosenblatt et al., 2016;<br>Durand et al., 2011.
